# Dynamic Modeling of the Sulfur Cycle in Urban Sewage Pipelines Under High-Temperature and High-Salinity Conditions

**DOI:** 10.3390/microorganisms13071534

**Published:** 2025-06-30

**Authors:** Zhiwei Cao, Zhen Xu, Yufeng Chen, Bingxuan Zhao, Chenxu Wang, Zuozhou Yu, Jingya Zhou

**Affiliations:** 1 Department of Agricultural Resources and Environment, College of Agriculture, Yanbian University, Yanji 133002, China; czw3543752471@163.com; 2 College of Geography and Ocean Sciences, Yanbian University, Yanji 133002, China; xuzhen@ybu.edu.cn; 3 Key Laboratory of Biology and Genetic Resources of Tropical Crops, Ministry of Agriculture, Institute of Tropical Bioscience and Biotechnology, Chinese Academy of Tropical Agricultural Sciences, Haikou 571101, China; chenyufeng@itbb.org.cn; 4 Department of Biological, Geological, and Environmental Sciences, University of Bologna, Via Sant’Alberto 163, 48123 Ravenna, Italy; gagt050251@gmail.com; 5 Department of Environmental Science, College of Geography and Ocean Sciences, Yanbian University, Yanji 133002, China; yueluan78719@163.com (C.W.); yuzuozhouyuzuozhou@126.com (Z.Y.)

**Keywords:** dynamic model, sulfur cycle, phased reaction process, sewage pipelines, corrosion

## Abstract

This study addresses the microbial corrosion of cement-based materials in coastal urban sewer networks, systematically investigating the kinetic mechanisms of sulfur biogeochemical cycling under seawater infiltration conditions. Through dynamic monitoring of sulfide concentrations and environmental parameter variations in anaerobic pipelines, a multiphase coupled kinetic model integrating liquid-phase, gas-phase, and biofilm metabolic processes was developed. The results demonstrate that moderate salinity enhances the activity of sulfate-reducing bacteria (SRB) and accelerates sulfate reduction rates, whereas excessive sulfide accumulation inhibits SRB activity. At 35 °C, the mathematical model coefficient “a” for sulfate reduction in the reactor with 3 g/L salinity was significantly higher than those in reactors with 19 g/L and 35 g/L salinities, with no significant difference observed between the latter two. Overall, high sulfate concentrations do not act as limiting factors for sulfide oxidation under anaerobic conditions; instead, they enhance the reaction within specific concentration ranges. The refined kinetic model enables prediction of sulfur speciation in tropical coastal urban sewer pipelines, providing a scientific basis for corrosion risk assessment.

## 1. Introduction

In modern societies, efficient, safe, and cost-effective sewerage networks play a vital role in safeguarding public health and preventing environmental pollution. Well-maintained sewer systems serve as a robust defense against drinking water contamination, hazardous sewer gases (H_2_S), and the risk of dispersion of various other pollutants. By ensuring the proper functioning of these sewerage networks, we can protect the well-being of communities and the ecological balance of our natural surroundings [1,2]. The sulfur cycle within the micro-ecosystem of the urban sewage pipeline network leads to the corrosion of cement-based materials, significantly reducing the service life of the sewage pipeline [3]. The severity of corrosion in urban sewage pipelines depends greatly on the neighboring environment, and in coastal cities, the marine environment is recognized as one of the most corrosive mediums. It has been reported that the consumption rate of oxygen in the sewage pipeline network increases when the temperature rises, which leads to the formation of an anaerobic environment and increases the formation rate of hydrogen sulfide gas under the reduction reaction of sulfur-reducing bacteria [3,4]. The combination of high temperatures, high levels, and exposure to coastal seawater poses serious corrosion risks for these essential infrastructure systems. Under such harsh conditions, urban sewage pipelines become highly vulnerable to deterioration and premature failure. The corrosion process of concrete surfaces within urban sewage pipelines involves a complex redox reaction, which is significantly influenced by microorganisms. In particular, understanding the corrosion mechanism related to the dynamic equilibrium process of the sulfur cycle is of utmost importance, especially in the environmental context of a coastal city.

Combined with the current results, the sulfur cycle’s dynamic response is key in assessing the effects of microorganisms on the surface corrosion of concrete materials [5]. In the early days, some scholars began to evaluate H_2_S generation in sewers using several models [5,6]; however, these models consider only the sulfur oxidation process or the sulfur reduction process alone, neglecting many details of biotransformation and therefore failing to fully represent the complete reaction process of the sulfur cycle. They also neglect the cross-influence of multiple factors in the anaerobic sewage pipeline. At the same time, studies have shown that the reaction process in which sulfate-reducing bacteria convert sulfate into sulfide, and the reaction process in which sulfur-oxidizing bacteria convert sulfide to S_0_ are integrated into a single reactor, which has practical guiding significance for exploring the sulfur cycle [7]. Next, the WATS model [8,9] in the sewer line was developed. This model is more complete in its biological representation compared to earlier models based on empirical expressions, and comprehensive environmental conditions within the sewage pipeline are taken into account in the research field of corrosion modeling. However, the existing models are models based on the transformation between aerobic and anaerobic environments [9,10]. Subsequent studies have revealed that the biofilm adhering to the inner wall surface of the upper section of the sewage pipeline network and the permanent sediment cover in the lower section create a strictly anaerobic environment [4,11]. These investigations have demonstrated that dissolved oxygen is virtually absent in this system. As sulfide is oxidized by free oxygen, the conversion of S_0_ is hindered. Further investigations have revealed that certain sulfate-reducing bacteria can utilize the formed S_0_ as an electron acceptor [9,12,13], thereby facilitating a more favorable sulfur cycle reaction. Understanding the influence of environmental impact factors on the sulfur cycle process is of paramount significance in this context. Previous research reveals that the mechanism governing the dynamic equilibrium process of the sulfur cycle under anaerobic and anoxic conditions within sewage pipelines remains unclear. This is especially true for the micro-ecological system of urban sewage pipe networks operating in the challenging environmental conditions of high temperature and high salinity in coastal areas. Limited studies have explored the microbial metabolism occurring within the biofilms under these conditions. To address these knowledge gaps and comprehensively understand the intricate processes at play, it becomes imperative to develop a comprehensive kinetic reference model specifically tailored for the anaerobic environments of high temperature and high salinity in urban sewage pipeline networks [14].

This study truly simulates the anaerobic environment of the urban sewage pipe network in high-temperature and high-salinity areas, and the changes in the internal environment and sewage index parameters of the anaerobic reactor by real-time monitoring. Then, based on the WATS model, this study investigates the dynamic responses of sulfate reduction rates and sulfide oxidation rates in high-temperature and high-salinity regions using the temperature and salinity of wastewater as environmental parameters. At the same time, this study optimizes the parameters of the existing sulfur cycle model to make it suitable for anaerobic environments. This study accurately describes the temporal and spatial variations of sulfur compounds during the transformation process in anaerobic reactors. It reveals the interactions among various factors influencing sulfur cycling and microbial activities, addressing the current lack of modeling for sulfur cycling in anaerobic environments. This enables us to develop effective strategies for managing corrosion in coastal cities. The improved kinetic model comprehensively [15] mitigates the harsh environmental challenges posed by coastal areas.

## 2. Materials and Methods

### 2.1. Design and Operating Conditions of Anaerobic Concrete Pipeline Reactors

This study encompassed the autonomous design and assembly of six parallel-operating anaerobic reactors, each with a volume of 76.93 L, employing a reaction principle that is akin to that of a UASB reactor [6,10,16]. Plexiglass covered the reactor, and the contact area was filled with glass glue to maintain a relatively well-sealed anaerobic environment. Based on the biological characteristics of the reactor, this study can be divided into five parts: anaerobic sedimentation layer, sewage layer, biofilm layer, gas atmosphere layer above the sewage liquid surface, and concrete material corrosion layer [15,17]. Reinforced sewage was introduced into the reactor. The production of biogas (mainly methane and carbon dioxide) causes the cycle of internal material, which is beneficial to the formation and maintenance of sludge particles. Some forming gas adheres to sludge particles in the sludge blanket, and the attached and unattached gases rise toward the top of the reactor. After the gases are released, the sludge particles will precipitate to the surface of the sludge blanket, and the attached and unattached gases will aggregate into the atmosphere at the top of the reactor. The gases produced by the reactor designed in this study were completely absorbed by the wall of concrete material.

The simulation was conducted to account for the unique environmental conditions of coastal cities, compared to a freshwater area. Consequently, the salinity gradients in the reactor were set at 3 g/L, 19 g/L, and 35 g/L. Temperature settings were carefully considered to encompass both regular temperatures and the long-term temperature variations experienced in coastal cities. As a result, two temperature gradients of 25 °C and 35 °C were established for this study. This method allows for a comprehensive investigation of the sulfur cycle and microbial activities under different temperature and salinity conditions.

### 2.2. The Inoculation of Sludge and Nutrition Medium

**Figure 1:**
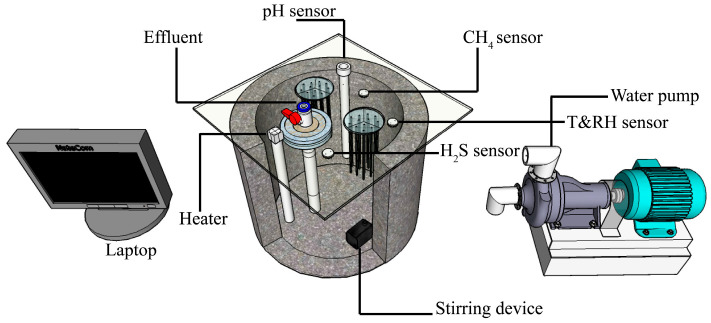
The setup of anaerobic concrete pipe reactor. The inside of the reactor is mainly composed of 9 components, which are laptop, effluent, heater, pH sensor, H_2_S sensor, CH_4_ sensor, water pump, T&RH sensor, and stirring device.

The sludge inoculated into the anaerobic concrete pipe reactor as shown in Figure 1 was taken from the Baishamen Wastewater Treatment Plant in Haikou City, Hainan Province. The Baishamen Wastewater Treatment Plant is located at the northernmost end of Haikou City. It is adjacent to Qiongzhou Strait, serving 700,000 people, and it is the most representative sewage treatment plant in the nearest living area from the seaside [18].

In essence, the wastewater in the Wastewater Treatment biological reactor is also a culture medium for microorganisms, and the enhanced wastewater which is configured must also follow some basic standard. In order to meet the microbial requirements for nutrients and maintain the normal growth of microorganisms in the activated sludge, pH buffer is often added into the microbial media, and acids or alkalis are added when there is industrial fermentation. This experiment considered the different nutrient requirements of different environmental microorganisms, in which the dosage of nitrogen and phosphorus was increased by the ratio of BOD:N:P = (350–500):5:1.

### 2.3. Real-Time Monitoring System

The inside of the reactor is composed of four parts, including a real-time temperature and humidity monitoring and acquisition system, a real-time gas monitoring and acquisition system, a real-time monitoring and acquisition system for sewage water quality index characteristics, and a stirring system. The real-time temperature and humidity monitoring and acquisition system is mainly composed of two parts: a YEE-YDH-606 heating rod (Manufacturer: Yuyao Yida Electrical Appliance Factory, Yuyao, China)and a Jianda Renke wall-mounted temperature and humidity sensor (Manufacturer: Jinan Jianda Renke Electronic Technology Co., Ltd., Jinan, China). The real-time gas monitoring and acquisition system is mainly composed of a CLE-0113-400 hydrogen sulfide gas sensor component (Manufacturer: Beijing Zhixin Sensing Technology Co., Ltd., Beijing, China). The sensor monitors a gas range of 0–1000 ppm and an accuracy of 0.09 ± 0.04 μA/ppm. The hydrogen sulfide gas above the liquid level of the sewage in the reactor mainly involves two flows, which are absorbed by the inner wall of the concrete material and re-flow into the sewage.

### 2.4. Collection of Basic Parameters

The real-time monitoring and acquisition system for sewage water quality index characteristics primarily focuses on monitoring the sewage index parameters of enhanced sewage within the reactor. These parameters include COD, ORP, pH, SO_4_^2−^ ion, S^2−^ ion, and VFA. The internal sewage sampling of the reactor was taken from the period after the reactor was stable. The specific sampling details of the experiment are summarized in Text S1 and Table S1. It is important to note that the dissolved oxygen (DO) concentration in the reactor remains below 0.2 mg/L.

### 2.5. Mathematical Modeling

In this study, the dynamically changing sulfur cycle model describes the sulfur conversion process in the liquid phase of the anaerobic sewage pipeline system [1], and simulation calculations were performed with the help of MATLAB/Simulink (Version R2023a, MathWorks, Natick, MA, USA) tools. The dynamic expression of this process was mainly derived from the models of Huisman and Hvitved-Jacobsen and was extended. However, this study did not involve a kinetic model of sulfidation and hydrogenation of the concrete corrosion layer.

The reaction process of sewage in the reactor can be divided into three biological processes, namely the hydrolysis, fermentation, and sulfur cycle reaction processes. Fermentation can be divided into two separate processes: the conversion of soluble COD to acetate and hydrogen and the fermentation to acetate and propionate. Acetate and propionate are the main constituents of volatile fatty acids (VFAs) [19] inside sewage pipes [7].

This kinetic model emphasizes the biochemical and physicochemical reactions taking place within the reactor, with a specific focus on comprehending and replicating sulfur cycling processes [20]. The comparison with the measured results led to the redefinition of the key parameters [21], with the primary objective of establishing correlations between these processes and environmental parameters. Table 1 lists the key biological processes and their kinetic expression formulas involved in the sewage reaction process inside the reactor; moreover, selected stoichiometries and kinetic constants can be found in Table 2. The slope of the measured concentration of hydrogen sulfide gas and time characterizes the sulfidation and hydrogenation rate of the concrete corrosion layer, and the calculated sulfidation and hydrogenation rate is simulated by the equation. Apply the ideal gas law to convert the oxidation rate of hydrogen sulfide from the unit of ppm s^−1^ to the flux for volume ratio conversion; that is, to determine the surface-specific oxidation rate of hydrogen sulfide gas, $F_{H_{2}S}[$g S·m^−2^ s^−1^].

### 2.6. Correlation Analysis: Variable Group A

The values of the biological model parameters in the sewage pipeline determined in the literature are suitable for most models [22]; however, achieving the best fit between the model predictions and field measurements requires manual adjustments to several key parameters of the model. In general, the stoichiometric parameters have higher first-order sensitivity than the kinetic parameters of biological models [23]. Model state variables may have greater impact on biological parameters. Different state variables will greatly interfere with model state variables. This will cause the output of the model to differ more, and therefore the sensitivity index will increase [24,25].

The Pearson correlation coefficient can be used to determine the degree of linear correlation between two variables, but it cannot measure the slope of linear and nonlinear relationships, and certainly cannot measure non-functional relationships. However, if correlation mining is performed using an algorithm without universality, the results are not complete, and many useful associations will be missed. The Maximum Information Coefficient (MIC) can measure the degree of correlation between two genes, capturing both linear or non-linear relationships [26]. As long as the two variables are not independent, their MIC coefficients are both 1, indicating higher accuracy than mutual information (MI). The MIC is an excellent calculation method for data correlation analysis. According to its nature, the MIC is universal, fair, and symmetrical.

The implementation of the MIC probability function, along with self-programming using Python software (version 3.11.5; https://www.python.org/), is given in Text S2 in the Supplementary Information.

## 3. Results and Discussion

### 3.1. Basic Parameter Indicators

This study conducted three cycles of repeated COD content measurement experiments to confirm the stability of the anaerobic concrete pipe interior in different groups, as shown in Figure S1. The changes in COD content before and after the three cycles are not very pronounced, and the decreasing trends are consistent, indicating that the environment inside the anaerobic concrete pipe has reached stability, allowing for the next phase of experimental research. Subsequently, effluent indicators were observed within one cycle. The specific parameters are shown in Table S2 in the Supplementary Information. During the reaction period, the oxidation–reduction potential of the wastewater in each reactor decreased from the initial value of about −200 mV to −100 mV, without any sharp changes. It was initially determined that the reactor was in a strictly anaerobic environment [1]. This is because the anaerobic environment inside the reactor provides good environmental conditions for the anaerobic microorganisms inside the reactor and supplies an electron donor to promote the oxidation reaction [27]. The specific factors will be described in the next section. At the same time, the pH value in each reactor fluctuated in a wavy-like pattern within the range of 7–8.3, showing an overall upward trend. The pH measured inside the reactor with higher temperature and salinity is always higher than that of the reactor with lower temperature and salinity, usually by no more than 0.5 pH units. The increase in pH can be attributed to the dissolution of corroded material slurry and powder into the sewage. However, the impact of temperature on pH was found to be negligible. Given the complexity of the reactions within the pipeline, further investigation is necessary. At the same time, the content of H_2_S gas in each reactor showed a wavy downward trend. The H_2_S gas above the liquid level inside the reactor continuously reacts with the inner wall of the reactor and penetrates into the concrete corrosion layer. Combined with ORP trend analysis, it was observed that the higher the ORP value in the reactor, the slower the sulfate reduction rate [28], and the production of H_2_S gas also gradually decreased.

### 3.2. Correlation Analysis: Variable Group B

To ensure the credibility of the biological model for sewage within the reactor, this study examines the interaction of various parameters, such as COD, VFA, SO_4_^2−^, S^2−^, and pH indicators. The MIC analysis results are shown as Table 3.

The MIC results in Table 3 indicate that the environmental state parameters in the reactor have a great interference on the interaction between the five index parameters of the sewage in the reactor. It is difficult to determine the optimal parameters in the biological model of the reactor under specific circumstances.

This study summarizes the interaction information among the five index parameters of sewage in the reactor, as shown in the Table S3, and sewage indicators with a maximum confidence coefficient of 0.4 and above are correlated.

Anaerobic hydrolysis and fermentation are the main processes that determine the quality and composition change of organic matter in the reactor (Q. H. Zhang et al., 2016). When the temperature was 25 °C, the correlation between the soluble COD and VFA in the wastewater of the reactor was 0.52, at a salinity of 19 g/L. It is shown that when the temperature is 25°C, the internal hydrolysis of the reactor may limit the fermentation and sulfate reduction reaction processes [2,13], and acid-producing microorganism aggregates compete with SRB for the fermentable substrate S_F_. When the temperature is 25 °C and the salinity is 19 g/L, the soluble COD and VFA in the reactor internal wastewater show a certain degree of correlation, which will be analyzed in the next section. At a temperature of 35 °C, and with salinity levels set at 3 g/L and 35 g/L, the soluble COD in the reactor’s wastewater exhibits a robust correlation with VFA. Notably, this correlation strengthens with the higher salinity level. Additionally, it is evident that the temperature of 35 °C represents the optimal active state for the system [28]. SRB will use a large amount of fermentable substrate S_F_ as the fermentation substrate, and it is suitable for the Monod model of SRB anaerobic fermentation based on the fermentable substrate S_F_. However, when the temperature is 35 °C and the salinity is 19 g/L, the soluble COD and VFA in the sewage in the reactor do not show a correlation, which will be analyzed in the next section.

The soluble COD and VFA in the reactor’s wastewater exhibit a relationship with S^2−^. However, the correlation among sewage index parameters within the dynamic model does not display a clear pattern in response to changing environmental factors. This suggests the universal nature of the sulfur reduction reaction process within the internal kinetics model of the reactor, which remains unaffected by environmental parameters in terms of modifying the sulfur reduction reaction process parameters. Based on the model proposed by Nielsen, the model parameters appear as pH dependence. However, it is worth noting that when the temperature was 25 °C with a salinity of 35 g/L, or when the temperature was 35 °C with a salinity of 3 g/L and 19 g/L, the SO_4_^2−^ concentration in the sewage within the reactor showed a strong correlation with the pH value. This shows the specificity of the sulfur oxidation model in the reactor [29], but the influence of environmental parameters on the modification of the reactor’s sulfur cycle sulfur–oxygen reaction process parameters cannot be ruled out. Based on the concept of charge balance in wastewater, a pH model of wastewater was established. The model determines the relationship between the pH and the water properties. The S^2−^ in sewage within the reactor showed a strong correlation with the pH value, with the pH being a key parameter determining the extent of sulfur-containing substance inhibition. It shows that the sulfur circulation system inside the reactor constitutes one of the main factors causing the pH change in sewage. Because this study does not involve the change of acid-base balance caused by the carbon cycle system [30], it will not be discussed in depth. As a result of VFA degradation, alkalinity generation is directly proportional to COD [31]. Therefore, there is a strong correlation between the soluble COD and the pH value of the wastewater in the reactor.

This study analyzed the interaction between the parameters of the wastewater indicators in the reactor from the results of the MIC. To precisely assess the applicability of the biological mathematical model within the reactor, it is essential to begin with the Pearson correlation analysis. This analysis will help determine the expression trends of each biological reaction process in the reactor [32], as illustrated in Table 4.

This study presents a comprehensive summary of the linear correlation among the five index parameters of the sewage within the reactor, as shown in Table S4. The magnitude of the absolute value of the Pearson coefficient indicates the strength of the linear correlation between these sewage index parameters. Positive and negative values signify positive and negative correlations, respectively, among these parameters.

At a temperature of 25 °C, with a salinity of 35 g/L, and at a temperature of 35 °C, salinities of 3 g/L and 19 g/L, the SO_4_^2−^ and pH of the sewage in the reactor show [19] a positive correlation [33,34]. However, the linear correlation is relatively weak, so it is in line with the nonlinear correlation of the sulfur oxidation reaction process parameters of the sulfur cycle model. Most biological mathematical models of sewage pipelines assume that sulfate is the non-restrictive matrix for the oxidation of sulfides [35], but when the sewage stays in the actual sewage pipeline in the city, sulfate is gradually deposited in the anaerobic sediment layer at the bottom of the sewage pipeline [1]. Sulfate may be the limiting substrate [36] in the oxidation of sulfides. In addition, temperature is one of the most important factors affecting the activity of sulfur-oxidizing bacteria. At a temperature of 25 °C, the activity of sulfur-oxidizing bacteria is limited [1,2], and the accumulation of sulfate at a salinity of 35 g/L will also limit the sulfuric acid reaction processes within the sewage. However, in the sulfur oxidation mathematical model of the sulfur cycle, the preliminary determination is more applicable under the above-mentioned temperature and salinity conditions, so further research is needed.

Based on these results, at a temperature of 25 °C with salinity levels of 3 g/L and 35 g/L, as well as at three salinity gradients at a temperature of 35 °C, the COD, VFA, and S^2−^ in the reactor’s wastewater demonstrated a negative correlation with each other. The correlation was weak, aligning with the parameter correlation observed in the sulfur reduction reactions of the sulfur cycle model.

Conversely, at a temperature of 25 °C and a salinity of 19 g/L, COD, VFA, and S^2−^ exhibited a positive correlation. However, this positive correlation was preliminarily attributed to potential data collection errors.

### 3.3. Biological Model

The model of the sulfur cycle in the internal sewage of the reactor includes the maximum sulfide productivity and sulfide oxidation rate of the biofilm surface area. By adjusting these mathematical model parameters, a favorable alignment is achieved between the simulated data and the measured data.

#### 3.3.1. Sulfur Reduction Model

**Figure 2:**
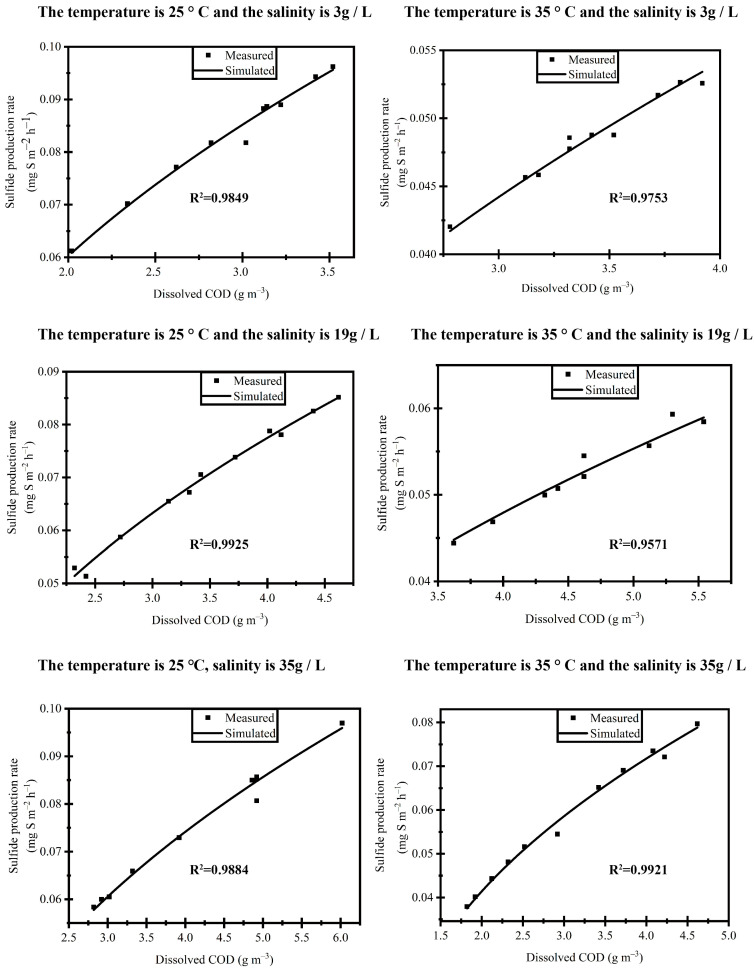
The fitting curves of SO_4_^2−^ and soluble COD in different pipeline reactors. The coefficient “a” is 0.37321 ± 0.00178, 1.32074 ± 0.00134, 0.05575 ± 6.4155 × 10^−18^, 0.23449 ± 0.00129, 0.44502 ± 0.00261, and 0.29622 ± 5.28517 × 10^−17^.

At the end of the reaction, the organic soluble COD of the sewage in the reactor was found to be lower than the hydrolysis rate X_S1_ of the granular matrix. As per the formula, this implies that after the organic soluble COD undergoes hydrolysis and converts into a small amount of nutrient matrix, it fails to provide sufficient energy for the metabolism of sulfate reducing bacteria. The reaction cycle is divided into two primary stages: the sulfate reduction reaction and the sulfuric acid reaction. To more accurately simulate the sulfate reduction rate within the coastal urban sewage pipeline and evaluate the reactions inside the reactor, this study focuses on the mathematical simulation of the sulfur reduction reaction process during the initial 12 h of the reaction cycle, as illustrated in Figure 2. The sulfate reduction rate within the reactor is precisely simulated using the exponential function of the soluble COD of the sewage in the reactor. Remarkably, no significant differences in the sulfate reduction rates were observed among the six reactors. The coefficient “a” in the mathematical simulation equation of the sulfate reduction reaction process in the sulfur cycle model of the reactor plays a crucial role in determining the rate of change in the sulfate reduction rate. However, it is important to note that when the organic soluble COD is lower than the hydrolysis rate X_S1_ of the granular matrix, the reaction cannot continue. Throughout this study, the sulfate reduction rate in all reactors exhibited a process of dynamic equilibrium change, signifying a balanced and stable reaction state. As the internal temperature and salinity of the reactor increase [20], the degree of fluctuation in the system tends to become more moderate. Simultaneously, the coefficient “a” in the mathematical simulation equation of the sulfate reduction reaction process decreases, leading to a relatively prolonged reaction stage for the sulfur reduction process within the reactor. This shows that higher temperatures and salinity levels contribute to a more stable and sustained sulfate reduction reaction. This study is based on the accelerated experiment [37]. The sulfate reduction rate in different reactors is larger than the results of field surveys in Nagasaki and Oga, Japan. The coefficient “a” exhibits a difference of approximately one order of magnitude. This result is relatively small when compared to the findings from exposing the biofilm surface in gravity sewers to low levels of oxygen [38]. However, it is essential to consider that the reactor in this study functions as an anaerobic closed system, and the presence of free ammonia and sulfide in the sewage inside the reactor can be toxic to microbial activity [29,39]. Consequently, the rate of sulfate reduction may exhibit a relatively decreasing trend.

At a temperature of 25 °C and varying salinity levels of 3 g/L, 19 g/L, and 35 g/L, the mathematical model coefficient “a” of the sulfate reduction reaction process in the different reactors does not exhibit a significant difference, and it follows an initial increase, followed by a decrease. This trend suggests that an appropriate increase in salinity enhances the activity of sulfate-reducing bacteria to a certain extent, leading to an improved sulfate reduction rate. However, excessive sulfide storage within the sewage can be toxic to the sulfate-reducing bacteria, resulting in the inhibition of their activity [40]. At a temperature of 35 °C, the mathematical model coefficient “a” of the sulfate reduction reaction process in the reactor with a salinity of 3 g/L is significantly higher than in the reactors with salinity levels of 19 g/L and 35 g/L. Furthermore, there is not a substantial difference in the mathematical model coefficient “a” of the sulfate reduction reaction process in the sewage of the reactors with salinity levels of 19 g/L and 35 g/L. Temperature plays a pivotal role in influencing the activity of microbial aggregates [22]. With an increase in temperature, the activity of sulfate-reducing flora is enhanced, leading to a certain degree of improvement in the sulfate reduction rate. However, the sulfate reduction rate coefficients in the reactors with salinities of 19 g/L and 35 g/L displayed abnormally small values, indicating that the sulfides produced in these reactors inhibited the expression of sulfate-reducing bacteria [41]. Interestingly, as the salinity increases, the internal reaction of the reactor tends to become more stable. However, there is a significant difference in the hydrogen sulfide gas within the reactor, particularly at a salinity of 3 g/L. Moreover, as the temperature inside the reactor rises, there is an observable trend of increasing hydrogen sulfide gas production. The solubility of hydrogen sulfide in water decreases with increasing temperature [33] and increasing salinity of the sewage. In a lower temperature environment within the pipeline reactor, more hydrogen sulfide produced during the sulfate reduction reaction dissolves into the water and cannot diffuse. On the other hand, when the salinity of the sewage is increased, it promotes the escape of hydrogen sulfide, leading to an increasing trend in the hydrogen sulfide content in medium-salinity and high-salinity environments. Moreover, in high-salinity situations, the hydrogen sulfide content of the gas shows a significant increase.

#### 3.3.2. Sulfuric Acid Model in Sewage

At a temperature of 25 °C and a salinity of 35 g/L, as well as at a temperature of 35 °C with a salinity of 3 g/L, there is a noticeable difference between the predicted sulfide concentration and the measured sulfide concentration in the reactor, leading to less-than-ideal fitting results. Moreover, the reaction order of sulfide concentration falls between 2 and 3, significantly exceeding the typical value of n1 (between 0.8 and 1.0) reported by Nielsen [29,31,42]. Similarly, when the temperature is 35 °C and the salinity is 19 g/L, the difference between the predicted sulfide concentration and the measured sulfide concentration in the reactor is also substantial. This indicates that when the sulfate concentration in the reactor is 19 g/L, the mathematical model of the sulfuric acid reaction process inside the reactor cannot neglect the influence of sulfate concentration.

The assessment of the sulfuric acid reaction is based solely on the apparent data. By applying linear regression to the concentration distribution of organic soluble COD, the reaction inside the reactor can be divided into two stages: COD dissolution and COD degradation. During the initial 12 h of feeding, the organic soluble COD in each reactor exhibits a decreasing-then-increasing pattern. These two phases, responsible for the change in organic soluble COD, are closely linked to the pH value of the sewage in the reactor, demonstrating a pH-dependent behavior. When the COD degradation rate is stronger than the COD dissolution rate, the pH decreases. However, the precise cause of this pH dependence remains unclear. Such phenomena are common in many enzyme-driven biological reactions, as each enzyme exhibits optimal activity at specific pH levels [43].

Within the closed anaerobic environment of the reactor, the oxidation of hydrogen sulfide gas leads to two outcomes. A portion of the biological sulfuric acid remains on the surface of the concrete pipe wall, causing corrosion, while another portion flows into the sewage, increasing the sulfide content in the wastewater. Sulfides can bind to iron in cells, inhibiting the respiration of bacterial organisms [44], and exhibiting toxicity to various microorganisms [45,46]. As a result of the inhibition of heterotrophic bacteria activity in the pipeline, the removal efficiency of organic soluble COD is reduced, and the reactor itself is in a change of biological dynamic equilibrium.

An increase in temperature within a certain range enhances the activity of heterotrophic microorganisms. However, it also results in an increased release of sulfide back into the effluent, which in turn reduces the removal efficiency of organic soluble COD. Notably, the sulfuric acid reaction rate in the reactor [23] is significantly higher than the sulfate reduction rate, indicating that the toxic effect of sulfide on the microorganisms within the reactor surpasses the increase in microbial activity at high temperatures [47].

#### 3.3.3. Sulfuric Acid Model in in Gas Atmosphere

The solubility of hydrogen sulfide gas in water decreases with increasing temperature [48] and also decreases [9] with increasing salinity in wastewater. The lower the internal temperature of the reactor, the more hydrogen sulfide gas generated during the sulfate reduction reaction dissolves into the sewage rather than escaping. When the salinity levels are 19 g/L and 35 g/L, the hydrogen sulfide gas content in the reactor is high, and it further increases significantly when the salinity is 35 g/L. In this study, the sulfuric acid rate inside the reactor was mathematically simulated using the formula. The coefficient k_p_ of the sulfuric acid reaction equation is 2.214 × 10^−4^, indicating that the hydrogen sulfide gas inside the reactor is already in a relatively saturated state, and further simulation calculations cannot be performed [14].

### 3.4. Model Validation

This study performed periodic simulations on a sulfur reduction model with a good fit in biological processes. The comparison between the fitted data and the simulated data revealed a difference of only 1% to 1.5%, confirming the credibility of the simulated data.

In this study, Principal Component Analysis (PCA) was performed on the basic wastewater indicators in the reactor, and the fermentation process [37], the sulfur reduction model, and the sulfur oxidation model of the biological process in the reactor demonstrated in this study were verified one by one, as shown in Table 5.

The correlation between COD and sulfide [13] is the strongest, and the main component contribution rate [49] is 91.751%. The correlation between COD and sulfide and the principal component is strong, and their correlation with the principal component is negative and positive, respectively, further confirming the applicability of the sulfur reduction model in sewage. However, the correlation between the basic sewage parameters in the reactor is weak, with main component contribution rates of only 62.866% and 53.008%. COD and VFA, as well as sulfate and sulfide, showed positive correlations with the principal components, although these correlations were weak. The relationship between sulfate and the main component indicates that a certain concentration of sulfate in the sewage can enhance the sulfur oxidation rate in the system.

## 4. Conclusions

Based on the observed changes in the wastewater in the anaerobic pipeline, we have enhanced the existing dynamic model to establish a dynamic model for sulfur cycling in anaerobic environments. This model captures the dynamic equilibrium changes between sulfur reduction and sulfur oxidation reactions throughout the reaction cycle. At the interfaces of liquid phase, gas phase, and biofilm, the basic parameters in the kinetic model serve to characterize the smoothness of the reactions within the sewage pipe reactor and the persistence of the sulfur cycling process.

The previous biological model developed for sewage pipelines had certain limitations, including the restriction imposed by S_F_ on the fermentation of SRB in the sewage. We observed that high-concentrations of sulfate in wastewater did not act as a limiting factor for the sulfur–hydroxide reaction. On the contrary, when sulfate was incorporated into the sulfur cycle process in the sewage pipeline, a certain concentration of sulfate actually promoted the sulfur–hydrogen reaction process in the wastewater.

When the internal temperature of the anaerobic concrete pipeline reactor was 35 °C, the coefficient of change of the sulfate reduction rate “a” was slightly lower than the average reported in the literature for middle-salinity pipelines and high-salinity pipelines (middle-salinity pipeline, a = 0.23449, high-salinity pipeline, a = 0.29622), indicating that the sulfate reduction model tends to be flat, and the sulfate reduction rate inside the pipeline reactor is stable.

When the internal temperature of the anaerobic concrete pipeline reactor was 25 °C, and the salinity ranged from 3 g/L to 35 g/L, the sulfate-reducing flora in the pipeline reactor exhibited the best active state. The kinetic model comprehensively considers the effects of temperature and salinity on microbial activity, and can better evaluate the corrosion of concrete pipelines by micro-ecosystems in sewage pipe networks in high-temperature and high-salinity areas, providing new insights into the microbial corrosion of sewage pipe systems in coastal areas.

Under high-temperature and high-salinity conditions, the dynamic modeling of the sulfur cycle in urban sewage pipelines is of great significance. From the perspective of the pipeline life cycle, this research helps to gain a deeper understanding of the mechanisms by which sulfur cycling affects pipeline corrosion, providing a scientific basis for pipeline material selection and corrosion prevention design. Moreover, through dynamic modeling, the generation and transformation of sulfides under different operating conditions can be predicted, which aids in optimizing pipeline operation and maintenance strategies and extending pipeline service life. Meanwhile, this study may also reveal the interactions between sulfur cycling and microbial communities, offering new insights for the ecological restoration and environmental management of sewage pipelines.

## Figures and Tables

**Table 1:** Summary of model processes and kinetic expressions.

SN	Process Reaction	Reference Component	Reaction Rate
Fermentation	FCOD → VFA	S_F_	$-q_{fe}\frac{S_{F}}{K_{fe}+S_{F}}\frac{K_{{NO}_{3}}}{K_{{NO}_{3}}+S_{{NO}_{3}}}\frac{K_{O_{2}}}{K_{O_{2}}+S_{O_{2}}}(X_{BW}+\varepsilon X_{Bf}\frac{A}{V})$
Sulfide generation using fermentable substrate	2FCOD + SO_4_ → H_2_S	$S_{H_{2}S}$	$r_{a}=a\sqrt{S_{F}+S_{A}+X_{S_{1}}}\;\frac{K_{O}}{K_{O}+S_{O}}\frac{K_{NO_{3}}}{K_{NO_{3}}+S_{NO_{3}}}\frac{A}{V}\alpha^{\left(T-20\right)}$
Sulfide oxidation	2O_2_ + H_2_S → SO_4_	$S_{{SO}_{4}}$	$r_{S(-\mathrm{II})}=K_{S(-\mathrm{II})}{\left(C_{S}\right)}^{n1}{\left(C_{O}\right)}^{n2}$
Concrete corrosion layer	2O_2_ + H_2_S → SO_4_	$S_{{SO}_{4}}$	$F_{H_{2}S}=\frac{d(p_{H_{2}S})}{dt}0.1013\frac{32}{R_{g}T_{abs}}\frac{V_{gas}}{A_{conc}}=[k_{p}0.1013\frac{32}{R_{g}}\frac{V_{gas}}{A_{conc}}]p_{H_{2}s}^{n}=k_{F}p_{H_{2}S}^{n}$

**Table 2:** Selected stoichiometries and kinetic constants.

Symbol	Definition (Unit)	Typical Value
*S*_F_	Fermentable, readily biodegradable substrate (g COD m^−3^)	
*S*_A_	Fermentation products (g COD m^−3^)	
a	Rate constant for sulfide formation (g^0.5^ m^−0.5^ h^−1^)	0.03
*X*_S1_		
*K*_O_	Saturation constant for DO (g O^2^ m^−3^)	0.01–0.5
*S*_O_	Dissolved oxygen (g O^2^ m^−3^)	
*K*_NO3_	Saturation constant for nitrate (g NO^3−^ N m^−3^)	
*S*_NO3_	Nitrate concentration in the bulk water phase (g NO^3−^ N m^−3^)	
α_w_	Temperature coefficient for heterotrophic, aerobic water phase processes (–)	1.07
α_f_	Temperature coefficient for aerobic biofilm processes (–)	1.05
*A*	Biofilm surface area (m^2^)	
*V*	Water volume (m^3^)	
*r*_s(–II)_	Rate of sulfide oxidation (g S m^−3^ day^−1^)	
*k*_S(–II)_	Rate constant (unit depends on the values of n1 and n2)	
*C*_S_	Concentration of dissolved sulfide (g m^−3^)	
CO	Concentration of DO (g m^−3^)	
*n* = *n*1 + *n*2	Reaction order (unit dependent on n1 and n2)	
*k*_S(II)c, pH_	pH-dependent rate constant for chemical sulfide oxidation ((g S m^−3^)^1−n1c^ (g O_2_ m^−3^)^−n2c^ h^−1^)	
*k*_H2Sc_	Rate constant for chemical sulfide oxidation of molecular sulfide, H2S ((g S m^−3^)^1−n1c^ (g O_2_ m^−3^)^−n2c^ h^−1^)	
*k*_HS-c_	Rate constant for chemical sulfide oxidation of ionic sulfide, HS–((g S m^−3^)^1−n1c^ (g O_2_ m^−3^)^−n2c^ h^−1^)	
K_a1_	The first dissociation constant for sulfide	
*k*_S(II)b, pH_	pH-dependent rate constant for biological sulfide oxidation ((g S m^−3^)^1−n1c^ (g O_2_ m^−3^)^−n2c^ h^−1^)	
*k*_S(II)b, pHopt_	Maximum rate constant for biological sulfide oxidation at the pH_opt_ value ((g S m^−3^)^1−n1c^ (g O_2_ m^−3^)^−n2c^ h^−1^)	
pH_opt_	Optimum pH value for activity of sulfide oxidation (–)	
*f_S_*_(II), pH_	Factor for relative pH dependency (–)	
ω*_S_*_(II)b_	Constant that determines the shape of the activity curve for sulfide oxidation versus pH (–)	
*μ*_Hw,NO3_	Maximum specific anoxic growth rate for heterotrophic biomass in the water phase (day^−1^)	2–6
*K*_Sw_	Saturation constant for readily biodegradable substrate in the water phase (g COD m^−3^)	0.5–2.0
*K*_1/2_	1/2-order reaction rate constant per unit area of biofilm surface (g NO_3_ N^0.5^ m^−0.5^ h^−1^)	6
*K*_sf_	Saturation constant for readily biodegradable substrate (g COD m^−3^)	

**Table 3:** The basic parameters of the wastewater in the reactor are the interactive information of soluble COD, VFA, sulfate, sulfur disulfide, and pH: Maximum Information Coefficient.

Maximum Information Coefficient	Low Salinity 25 °C	Low Salinity 35 °C	Medium Salinity 25 °C	Medium Salinity 35 °C	High Salinity 25 °C	High Salinity 35 °C
COD	0.99	0.47	0.99	0.47	0.47	0.99
VFA	0.29	0.47	0.52	0.52	0.2	0.99
Sulfate	0.29	0.47	0.52	0.52	0.47	0.2
Sulfide	0.47	0.47	0.99	0.47	0.52	0.47

**Table 4:** The basic parameters of the wastewater in the reactor include the linear relationships among soluble COD, VFA, sulfate, sulfur disulfide, and pH, as determined by Person correlation.

Pearson Coefficient	Low Salinity 25 °C	Low Salinity 35 °C	Medium Salinity 25 °C	Medium Salinity 35 °C	High Salinity 25 °C	High Salinity 35 °C
COD	−0.88	−0.89	−0.92	−0.58	−0.4	−0.9
VFA	−0.56	−0.81	−0.86	0.049	−0.1	−0.82
Sulfate	0.22	−0.023	−0.21	0.47	0.12	−0.15
Sulifde	0.85	0.42	−0.97	−0.31	0.77	0.46

**Table 5:** Principal Component Analysis of COD and VFA, COD and sulfide, and sulfate and sulfide among the basic sewage parameters of the model samples.

PCA	COD and VFA		COD and Sulfide		Sulfate and Sulfide	
Principal component contribution	62.866		91.751		53.008	
Single sample	COD	VFA	COD	Sulfide	Sulfate	Sulfide
Sample principal component	0.793	0.793	−0.958	0.958	0.687	0.687

## Data Availability

The original contributions presented in this study are included in the article/supplementary material. Further inquiries can be directed to the corresponding author.

## Supplementary Materials

The following supporting information can be downloaded at https://www.mdpi.com/article/10.3390/microorganisms13071534/s1. Table S1. The collection cycle of the internal sewage of the reactor. Table S2. The basic parameters of the sewage in the reactor are ORP, pH, and H_2_S ranges as follows. Table S3. Sewage indicators with a certain correlation inside the reactor are selected and summarized, and the maximum confidence coefficient between the sewage indicators of 0.4 and above is correlated. Table S4. Pearson coefficients between the five index parameters of the sewage in the reactor. Figure S1. Concrete sewage pipeline reactor water quality COD.
