# Supplementary material for: Dynamic Modeling of the Sulfur Cycle in Urban Sewage Pipelines Under High-Temperature and High-Salinity Conditions
Source: Microorganisms. 2025 Jun 30;13(7):1534. doi: 10.3390/microorganisms13071534 (PMC12299050; doi:10.3390/microorganisms13071534)
Supplement: Supplementary file 1 [file microorganisms-13-01534-s001.zip › microorganisms-3582320-supplementary.pdf]

Text S1

Sampling was conducted at regular intervals to analyze the volatile fatty acid (VFA) and the consumption rates of COD in the liquid phase. As the concentration of the above parameters in the system stabilized and no longer exhibited significant changes, a pseudo-steady state was reached. Following this, a field cycle test was performed every 168 hours to validate the system's performance over time. Additionally, a 120-day multi-cycle behavior study was carried out to gain comprehensive insights into the long-term behavior of the system and to validate the accuracy of the sulfur cycling model.

ORP was measured using a METTLER LE501 redox electrode. The pH sensor was measured using an E-201-CPH electrode. The stirring system is mainly composed of two HJ-1500 submersible pump components. Two submersible pumps are placed diagonally at the low end of the reactor. The COD in the wastewater of the reactor is mainly directed to soluble COD in the wastewater, and the wastewater is filtered through a 0.45μm polytetrafluoroethylene filter membrane before sampling. SO_4^{2-} ion, S^{2-} ion, total nitrogen, and total phosphorus were all measured by SmartChem140, and all water samples were filtered through a 0.45μm polytetrafluoroethylene filter membrane. A large amount of organic matter in the sewage will affect the determination of SO_4^{2-} ions in the sewage. The filtered water samples need to be further adsorbed with activated carbon. It was pre-treated with 0.1 M formic acid and then analyzed using gas chromatography. During transport to the laboratory, all field samples are stored in ice boxes and kept refrigerated until analysis. VFA substances (acetic acid, propionic acid, butyric acid, and valeric acid) were measured on an Agilent-7890B Agilent gas chromatograph at 100 ℃ using a RTX-WAX column (30. 0 m * 0. 25 mm * 0. 25 μm length * ID * film) measurement.

Text S2

The implementation of Maximum Information Coefficient probability function and self-programming of Phython software are as follows:

$$I(x,y) = \int p(x,y) \log_2 \frac{p(x,y)}{p(x)p(y)} dxdy$$

```
import pandas as pd
import matplotlib.pyplot as plt
from minepy import MINE
```

```
import matplotlib.pyplot as plt
import numpy as np
wine = pd.read_excel('wine.xlsx')
def MIC_matirx(dataframe, mine):
    data = np.array(dataframe)
    n = len(data[0, :])
    result = np.zeros([n, n])
    for i in range(n):
        for j in range(n):
            mine.compute_score(data[:, i], data[:, j])
            result[i, j] = mine.mic()
            result[j, i] = mine.mic()
    RT = pd.DataFrame(result)
    return RT
mine = MINE(alpha=0.6, c=15)
data_wine_mic = MIC_matirx(wine, mine)
data_wine_mic
import seaborn as sns
import matplotlib.pyplot as plt
import matplotlib.pyplot as plt
def ShowHeatMap(DataFrame):
    colormap = plt.cm.RdBu
    ylabels = DataFrame.columns.values.tolist()
    f, ax = plt.subplots(figsize=(14, 14))
    ax.set_title(' HeatMap')
    sns.heatmap(DataFrame.astype(float),
                cmap=colormap,
                ax=ax,
                annot=True,
                yticklabels=ylabels,
```

```
                xticklabels=ylabels)
plt.show()
```

Tab.S1 The collection cycle of the internal sewage of the reactor

Sewage index	ORP	COD	pH	VFA	SO_4^{2-}	S^{2-}	Total phosphorus	Total nitrogen
Monitoring cycle	Every day	Initial 12h / day	Per hour	Every day	Initial 12h / day	Initial 12h / day	Initial 12h	Initial 12h

Tab.S2 The basic parameters of the sewage in the reactor are ORP, pH, and H_2S ranges as follows

Reactor / indicators	Low salt 25 °C	Low salt 35 ℃	Medium salt 25 ℃	Medium salt 35 ℃	High salt 25 ℃	High salt 35 ℃
ORP	-178.5~ -109.5	-192.5~ -139.5	-198.5~ -114.5	-168.5~ -123.5	-196.5~ -115.5	-181.5~ -133.5
pH	7.8~7.21	7.46~7.07	8.09~7.75	8.11~7.8	8.12~7.56	8.25~7.89
H_2S	1~8	4~16	18~81	43~114	52~167	113~204

Tab.S3 Sewage indicators with a certain correlation inside the reactor are selected and summarized, and the maximum confidence coefficient between the sewage indicators of 0.4 and above is correlated.

Maximum Information Coefficient	Low salt 25°C	Low salt 35°C	Medium salt 25°C	Medium salt 35°C	High salt 25°C	High salt 35°C
COD&VFA	0.29	0.52	0.52	0.29	0.29	0.99
COD&Sulfate	0.47	0.47	0.99	0.29	0.47	0.29

VFA&Sulfate	0.29	0.2	0.29	0.99	0.47	0.47
Sulfate&Sulifde	0.29	0.47	0.13	0.99	0.99	0.29
pH&Sulifde	0.47	0.47	0.99	0.47	0.52	0.47
COD&pH	0.99	0.47	0.99	0.47	0.47	0.99

Tab.S4 Pearson coefficients between the five index parameters of the sewage in the reactor

Pearson Coefficient	Low salt 25°C	Low salt 35°C	Medium salt 25°C	Medium salt 35°C	High salt 25°C	High salt 35°C
COD&VFA	0.26	0.8	0.75	0.68	-0.16	0.92
COD&Sulfate	0.11	0.24	-0.15	-0.01	-0.44	0.44
VFA&Sulfate	-0.55	0.02	0.26	0.2	0.64	0.55
Sulfate&Sulifde	0.06	0.078	0.17	-0.48	0.64	-0.34
pH&Sulifde	0.85	0.42	-0.97	-0.31	0.77	0.46
COD&pH	-0.88	-0.89	-0.92	-0.58	-0.4	-0.9

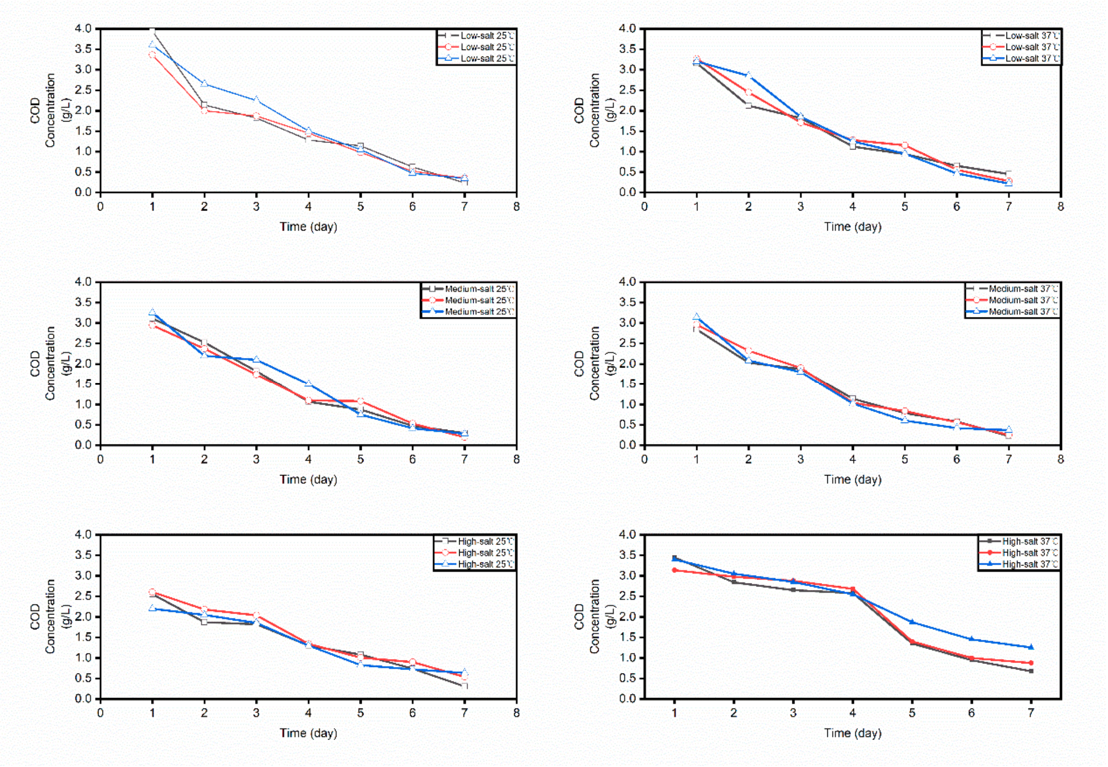

Fig.S1 Concrete sewage pipeline reactor water quality COD
